# A Mathematical Simulation of Copper and Nickel Ions Separation Using Prepared Nanocellulose Material

**DOI:** 10.3390/membranes13040381

**Published:** 2023-03-27

**Authors:** Saad Aljlil

**Affiliations:** Institute of Water Management & Treatment Technologies, King Abdulaziz City for Science and Technology (KACST), Riyadh 11442, Saudi Arabia; saljlil@kacst.edu.sa

**Keywords:** copper ions, nickel ions, nanocellulose, pore diffusion model, mass balances

## Abstract

Environmental risks can arise from the existence of heavy metals in wastewater and their land disposal. To address this concern, a mathematical technique is introduced in this article that enables the anticipation of breakthrough curves and the imitation of copper and nickel ion separation onto nanocellulose in a fixed-bed system. The mathematical model is based on mass balances for copper and nickel and partial differential equations for pore diffusion in a fixed bed. The study evaluates the impact of experimental parameters such as bed height and initial concentration on the shape of the breakthrough curves. At 20 °C, the maximum adsorption capacities for copper and nickel ions on nanocellulose were 5.7 mg/g and 5 mg/g, respectively. The breakthrough point decreased with increasing solution concentration at higher bed heights, while at an initial concentration of 20 mg/L, the breakthrough point increased with bed height. The fixed-bed pore diffusion model showed excellent agreement with the experimental data. The use of this mathematical approach can help alleviate the environmental hazards that arise from the presence of heavy metals in wastewater. The study highlights the potential of nanocellulose as a material for membrane technology, which can effectively address these risks.

## 1. Introduction

Heavy metal ions in wastewater pose a significant environmental risk due to their toxicity and non-biodegradability [1]. Therefore, removing heavy metals from wastewater is essential to mitigate the environmental problems associated with land disposal of contaminated wastewater. Several techniques are available for eliminating heavy metals from wastewater, such as membrane filtration, ion exchange, precipitation, adsorption, and co-precipitation/adsorption. Activated carbon is a commonly used adsorbent [1] and a highly effective technique for treating effluent-containing heavy metals via adsorption. Despite its widespread application in the water and wastewater treatment industries, activated carbon is still considered an expensive material [1].

Various adsorbents, including a powder derived from the shells of solemn-vaillant snails [2], were utilized to capture heavy metal ions. The research findings indicate that solemn-vaillant snail shell powder facilitated the removal of lead, copper, and cobalt by 96.5%, 96.7%, and 96.7%, respectively.

Khamseh et al. [3] investigated the adsorption of thorium (IV) ions by a protonated orange peel in a batch system using response surface methodology. They found that optimal conditions resulted in a maximum thorium uptake rate of 183.95 mg/g by evaluating the effects of initial thorium concentration, pH, and biosorbent dosage. The pseudo-second-order model was a good fit for the data from the experiment. After four hours, equilibrium was reached. Thermodynamics studies show that thorium can bind to protonated orange peel on its own and that this process releases heat. Industrial wastewater containing lead, copper, and nickel ions can be treated with granular activated carbon produced from palm kernel shells with a high metal adsorption capacity. Specifically, lead has an adsorption capacity of 1.337 mg/g, a copper capacity of 1.581 mg/g, and a nickel capacity of 0.130 mg/g [4]. Another study used Fe_2_O_3_ carbon foam with 3.62% iron content to remove heavy metals such as chromium, copper, and nickel from wastewater. The double exponential model showed that 6.7, 3.8, and 6.44 mg of heavy metals could be removed per gram of Fe_2_O_3_ powder [5]. African palm charcoal also effectively removed copper and nickel from industrial wastewater, with 92.01% and 90.41% removal probabilities, respectively [6]. Additionally, the adsorption capacity of copper from acidic wastewater using natural and calcined zeolite was studied. It was found that calcined zeolite had a 14.55% higher copper adsorption capacity than natural zeolite without any treatment [7].

Khamseh et al. [8] investigated thorium biosorption through an orange peel in a fixed-bed column, including the modeling of breakthrough processes and process parameters. Experiments with varying sorbent diameter, flow rate, bed height, and feed inlet concentration were conducted. The results demonstrated that the breakthrough point decreased with a decreasing bed height, increasing the feed inlet concentration, and increasing the flow rate, whereas the sorption capacity increased with a decreasing bed height and flow rate. The greatest sorption capacity of 87.7 mg/g was observed for sorbents with a diameter between 0.4 and 0.8 mm. The experimental data were analyzed using the Thomas, Yoon–Nelson, and Modified Dose-Response (MDR) models, with the MDR model providing the best fit.

Hajeeth et al. [9] conducted an investigation using cellulose graft acrylonitrile as an adsorbent for removing heavy metal ions, specifically copper and nickel, from aqueous solutions via adsorption. The effects of pH, contact time, and initial adsorbent dose on the metal ion adsorption capacity were studied. It was observed that the amount of heavy metal ions adsorbed increased with an increased shaking time, adsorbent dose, and alkaline pH. The optimal pH and adsorbent dose for Cu(II) and Ni(II) were 5.0 and 4.0 g, respectively. Lombardo and Thielemans [10] investigated the adsorption of various compounds onto nanocellulose surfaces, including natural binding proteins and various water pollutants such as heavy metal ions, dyes, and pharmaceuticals. They found that the adsorption process on nanocellulose surfaces could be explained as an interaction between charges with opposite signs.

Bertish and Fischer [11] investigated nanocellulose adsorption and interfacial structure at fluid interfaces. The researchers discovered that the adsorption of nanocelluloses at oil-water interfaces promotes the formation of biocompatible and stable Pickering emulsions. Madivoli and his colleagues [12] have pointed out that cellulose could be a cheap, renewable, and plentiful raw material for making filter membranes that clean water. Additionally, cellulose has a number of functional groups that make it possible to change it chemically to make active surfaces. They isolated cellulose from two readily available biomasses, Eichhornia crassipes and Cyperus papyrus, via the soda process and peracetic acid bleaching. In addition, the esterification of cellulose by citric acid was confirmed by analyzing the FT-IR absorption of the carbonyl functional group of an ester after cellulose nanofibrils were mixed with citric acid.

Adsorption commonly removes Ni^2+^ ions from agricultural wastes using bacterial biomass and activated carbon [13]. Ni^2+^ ions could be taken up by activated carbons made from different organic materials at a rate of 5.8 mg/g and bacterial biomass at a rate of 5.7 mg/g. Using two parameters (the initial concentration and bed height) to determine the effect on the breakthrough curve, Himanshu [14] examined the adsorption of heavy metals on an activated carbon fixed-bed column. They discovered optimal conditions occurred when initial concentrations were low and bed heights were high. As stated, wastewater treatment utilizes activated carbon to remove heavy metal ions. However, regenerating is costly and inefficient because it loses 5% of its adsorption capacity, making alternatives such as coagulation and biology more prevalent [15,16]. Some researchers are even investigating inexpensive substitutes for activated carbon, which can absorb heavy metals from wastewater. Since these methods are more effective at adsorbing heavy metals, alternative adsorbents have been used in some methods [16]. Nanocellulose derived from the palm is a popular adsorbent material because it is non-toxic, readily available, and inexpensive. Therefore, there remains a need for locally accessible and cost-effective sorbents, such as natural materials.

Even though it is not the main goal, nanocellulose’s unique properties make it potentially suitable for membrane applications. Findings from a study modeling copper and nickel ion adsorption can inform the design of nanocellulose membranes for heavy metal wastewater removal. The field of membrane technology, which includes numerous studies on nanocellulose, is rapidly expanding. Nanocellulose membranes have high permeability and good mechanical properties, making them effective water filters. Additionally, nanocellulose improves composite membranes’ mechanical strength, thermal stability, and permeability and can enhance existing membranes’ performance as a coating. Due to its low cost, biodegradability, and unique properties, nanocellulose has significant potential in membrane technology, prompting ongoing research into new applications, such as nitrocellulose-based composite membranes and coatings.

The nanocellulose membrane used in desalination applications was fabricated by Saud et al. [17] in their research. Nanocellulose is one of the most promising environmentally friendly materials for creating membranes because of its biodegradability, renewability, abundance, ease of modification, and superior mechanical properties. This makes it an excellent candidate for use in membranes. Additionally, the use of membranes composed of various nanocellulose forms, such as bacterial nanocellulose, cellulose nanocrystals, and nanofibrils, is also investigated. The analyses conducted in these studies demonstrated that membranes based on nanocellulose perform exceptionally well in desalination applications.

The research carried out by Tan et al. [18] offered a new perspective on nanocellulose-based membranes that can be manufactured. Since nanocellulose is both biodegradable and inexpensive, it has seen a lot of use in membrane research and development over the past decade. They concluded that incorporating nanocellulose with inorganic nanomaterials increased the pore size, porosity, and superhydrophilicity of the membrane. This led to a high water flow rate and selectivity, both of which are good for commercialization in the future. 

The work of Liu et al. [19] fabricated an ultrathin graphene oxide (GO) layer on a cellulose nanofiber (CNF) membrane to attain a crosslinker-free, robust, layered membrane with synergistic water flux and separation performance. Membranes exhibited a negative surface zeta potential, a significantly high water flux, and a higher water flux than a commercial reference membrane with a comparable water flux. Furthermore, the membranes rejected >90% of positively and negatively charged dyes via electrostatic interaction, hydrophobic interaction, and molecular size exclusion.

Jaffar et al. [20] provided insights into the potential of nanocellulose as a matrix or additive to improve membrane performance in water filtration, environmental remediation, and the development of pollutant sensors and energy devices. From the point of view of membrane technology, the unique mechanical strength, high crystallinity, tunable surface chemistry, and anti-fouling behavior of nanocellulose’s structural and nano-dimensional properties are very appealing. So, the chance has come to use these properties to make nanocellulose-based membranes that can be used in environmental applications. 

Rana et al. [21] extensively researched biocompatible membranes from novel cellulose-based materials. Cellulosic materials, particularly cellulose nanofibres, cellulose nanocrystals, bacterial nanocellulose, and various cellulose derivatives, have been found to increase the membrane’s salt-removal capability and water permeability through their increased porosity, pore size, and hydrophilic character. They concluded that cellulosic membranes’ potential in water desalination was as crucial as the fabrication steps in empowering the economy.

Overall, nanocellulose has significant potential in membrane technology due to its unique properties, low production cost, and biodegradability. As such, researchers continue to explore new ways to utilize nanocellulose in membranes, including developing novel nanocellulose-based composite membranes and coatings.

The primary objective of this investigation was to evaluate the efficacy of Saudi palm-derived nanocellulose in eliminating copper and nickel ions from wastewater via a fixed-bed methodology. Breakthrough curves were measured using a small laboratory column to validate the proposed approach. Various initial solution concentrations and bed heights were tested to conduct these measurements.

The significance and novelty of this study are primarily attributable to using a novel source for extracting nanocellulose from waste palm leaves in Saudi Arabia as an adsorbent for removing copper and nickel ions from wastewater. This new technique is good for the environment and costs less than other ways to make nanocellulose, which is used to remove heavy metals from wastewater. Previous studies used waste paper, cotton, and sugarcane bagasse as sources of nanocellulose. On the other hand, this study focuses on new source material to show its uniqueness.

This study is notable because it developed a mathematical model to simulate and predict the breakthrough curves for the adsorption of copper and nickel ions from wastewater onto prepared nanocellulose biosorbents using a fixed-bed system. The model’s combination of partial differential equations for pore diffusion and mass balances for copper and nickel in a fixed bed yields invaluable insights into the adsorption process. Testing the model with various experimental parameters increases its significance.

## 2. Materials and Methods

### 2.1. Nanocellulose as an Adsorbent

**Figure 1:**
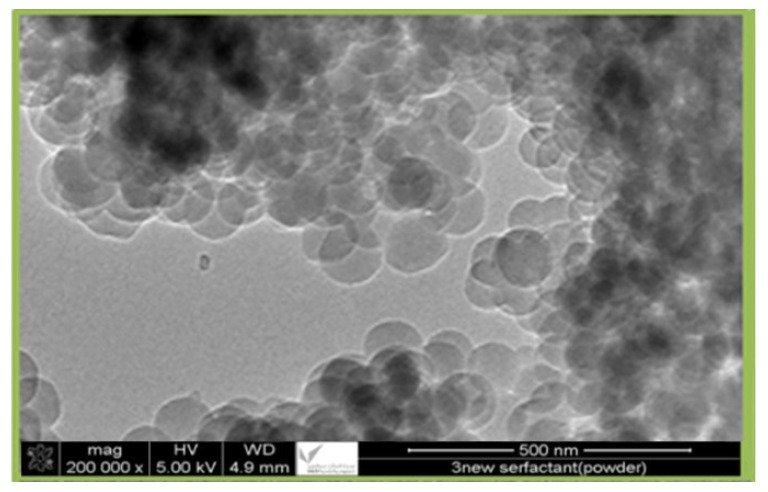
TEM of the nanocellulose (200,000×).

Waste palm leaves from Al-Karaj City, Saudi Arabia, were ground for six hours at 250 rpm in a planetary ball mill to produce a 65-micron powder. The powder was combined with distilled water and sonicated for 70 min using an SFX550 (Sonifier, Suwanee, GA, USA) at 480 W (Sonifier, Suwanee, GA, USA). Sulfuric acid was then slowly added to the mixture while being heated to 90 degrees Celsius. The mixture was centrifuged for 20 min at 3000 rpm, and the residue was filtered and washed multiple times with deionized water to neutralize the pH. Lignin was then removed through a four-hour hydrolysis process at 90 °C using 100 g of distilled water and 5 g of potassium hydroxide. The residue was then filtered and washed with deionized water to ensure its purity and achieve a neutral pH. In this method, cellulose is treated with a sodium chlorite solution (4 g/100 g water) to remove amorphous cellulose, and the solution is stirred for six hours at a constant temperature (80 °C). The final product was filtered and neutralized by repeated deionized water washings. The residue was then dried in an oven at 90 °C until it reached a constant weight, after which 50% sulfuric acid by weight was added at 50 °C for 70 min. The suspension was then centrifuged at 3000 rpm for 20 min, sonicated for 20 min at 480 W, centrifuged at 3000 rpm for 20 min, and filtered. Figure 1 displays the transmission electron microscopy (TEM) image (200,000×) of nanocellulose, obtained by collecting the precipitate and drying it in an oven at 50 °C for 4 hours. The resulting nanocellulose had an average size of 85 nm.

### 2.2. Preparation of the Copper and Nickel Ion Samples

Separate stock solutions (Cu and Ni) with a concentration of 1000 mg/L were prepared for each metal. The stock solutions were then diluted to the experiment’s required concentrations, ranging from 20 to 100 mg/L. After preparation, an atomic absorption spectrometer was used to analyze the initial concentration and diluted samples (at various time intervals) of metal ions (PerkinElmer Inc., AAnayst 700, Waltham, MA, USA).

### 2.3. Measurements of Metal Ions Concentration

For the purpose of measuring the concentration of metal ions in unknown samples, calibration curves were derived from solutions of known metal concentrations (copper or nickel). The solutions for the calibration curves had concentrations of 5, 10, 15, 20, 25, and 30 mg/L. The curve should be linear and demonstrate a linear relationship between concentration and absorbance.

### 2.4. Experimental Set-Up

**Figure 2:**
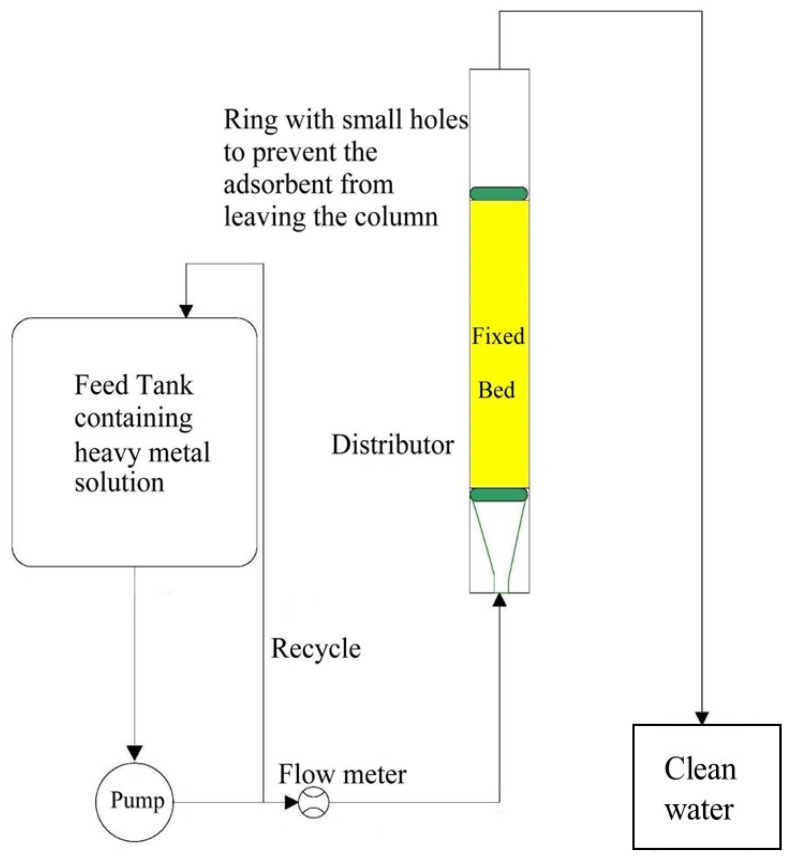
Sketch of the fixed-bed process.

**Figure 3:**
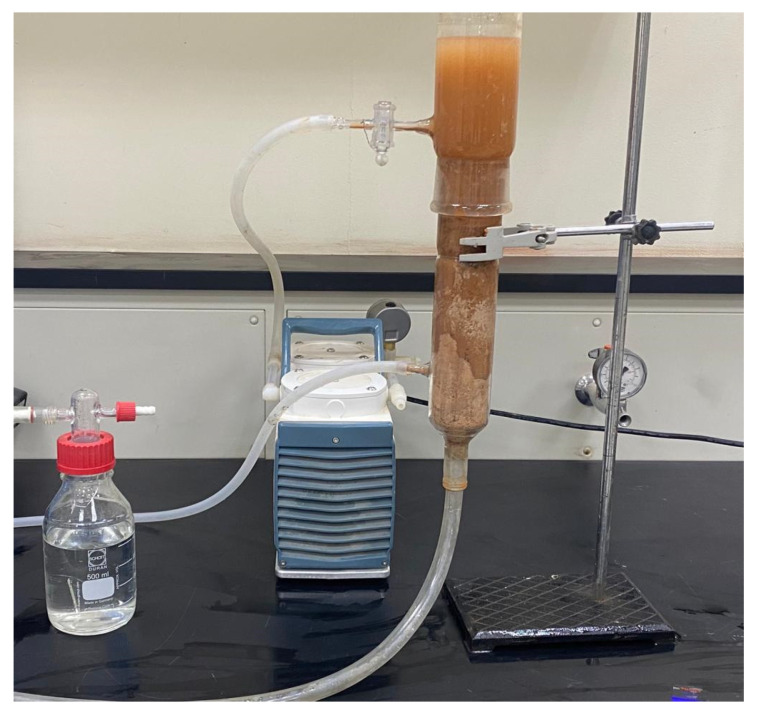
Showing the set-up of a fixed bed for the experiments.

To study the parameters affecting the breakthrough curve, a packed bed column made of plexiglass was created (i.e., initial concentration and bed height). The fixed bed consists of a 2-cm-diameter cylindrical tube containing a nanocellulose fixed bed. Utilize the long funnel to load nanocellulose into the tube. A distributor is located at the bottom of the tube to distribute the feed solution uniformly throughout the packed bed. A small, perforated copper disk is attached to the top of the packed bed to prevent the escape of sorbent particles. Using a Master Flex pump (Cole Parmer Instrument Co., Vernon Hills, IL, USA), dispense the solution from the 12 L tank through the packed bed at the desired flow rate indicated by the calibrated flow meter. Collect 15 mL samples from the outlet of the column bed at various time intervals and measure the concentration of unadsorbed metal ions in the column. The process of adsorption continues until the fixed bed is saturated with metal ions that have been adsorbed. As a feed solution, a metal ion solution was carried on each run. As depicted in Figure 2 (a schematic diagram of the fixed bed process), Figure 3 depicts the experimental setup employed for this study.

## 3. Results and Discussions

### 3.1. Nanocellulose Characterizations

#### 3.1.1. Transmission Electron Microscopy (TEM)

The prepared nanocellulose samples were observed using transmission electron microscopy (TEM), JEM-2100F, at 200 kV acceleration voltage. Usually, samples are sonicated in ethanol for six hours, and then they are placed on grids that have been coated with carbon and left to dry overnight. This step makes sure that the sample is spread out evenly across the grid and that everything is ready for imaging. Figure 1 is a TEM image of prepared nanocellulose at a magnification of 200,000×, with an average size of 85 nm.

#### 3.1.2. Fourier Transform Infrared Spectroscopy (FT-IRS)

**Figure 4:**
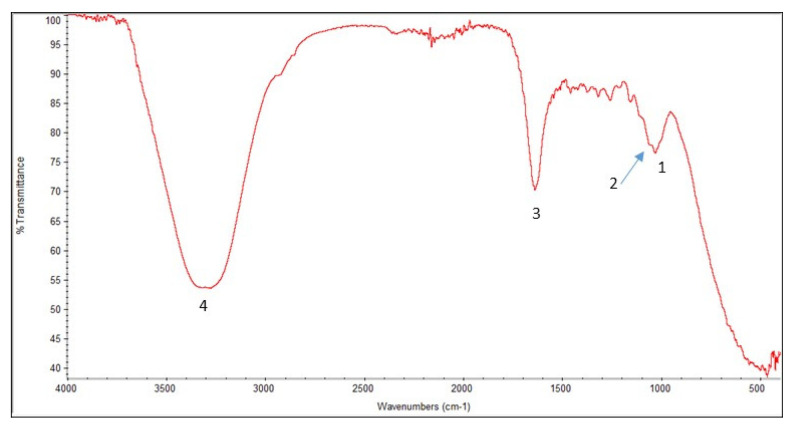
FT-IR spectrum of the nanocellulose. Peak 1 (1027 cm^−1^): C-O stretching in cellulose. Peak 2 (1164 cm^−1^): C-O stretching in cellulose and hemicellulose. Peak 3 (1730 cm^−1^): C=O stretching in hemicellulose. Peak 4 (3000–3399 cm^−1^): CH stretching in cellulose, hemicellulose.

In the wavelength range of 4600–400 cm^−1^, FT-IRS analyses were conducted using a mixture of 4 mg prepared nanocellulose powder and 200 mg KBr. Co-adding individual scans obtained the spectra with an FT-IRS device (ALFUAS, BRUKER type) with a scan resolution of 4 cm^−1^. Figure 4 depicts the peaks at 1027 to 1730 cm^−1^ and 3000 to 3399 cm^−1^ in the FT-IR spectrum of the scanned nanocellulose sample, indicating the presence of cellulose bonds. More details are in Figure 4.

#### 3.1.3. Zeta Potential

**Figure 5:**
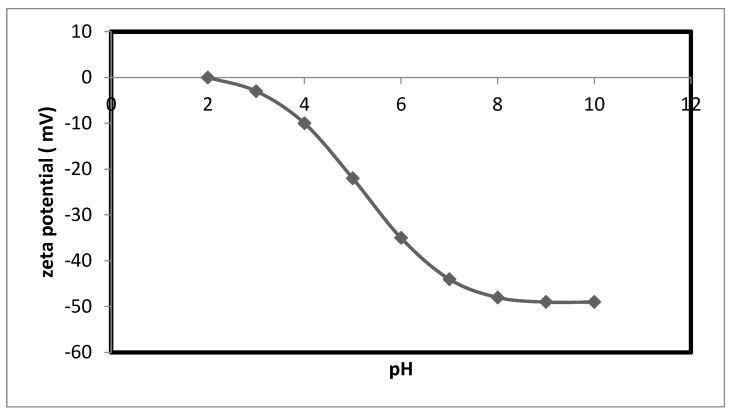
Variation of zeta potential versus pH for the nanocellulose.

Brookhaven Instruments Corporation, a company specializing in the manufacture of industrial equipment, produces Zeta Potential Analyzers. In this study, the instrument was utilized to determine the alteration in zeta potential and surface charge of the prepared nanocellulose. Furthermore, the correlation between the shift in zeta potential and pH was analyzed to determine the adsorbent’s surface charge. These findings are presented in Figure 5.

In order to figure out the surface charge of this adsorbent, the variation of the zeta potential of the nanocellulose with respect to pH has been studied. Figure 5 shows what the study found about how the zeta potential changed as a function of pH. The pH of the solution has an effect on the amount of copper and nickel ions that are adsorbed onto nanocellulose. The charge that is on the surface of the nanocellulose shifts depending on the pH, which causes differences in the amount of adsorption that takes place.

After the pH has been raised, the surface of the nanocellulose will have a negative charge, making it possible for it to adsorb the positive ions of copper and nickel.

So, the positively charged copper and nickel ions stick to the negatively charged nanocellulose because the negative charges and the positively charged copper and nickel ions are attracted to each other electrostatically. This force causes the negatively charged ions to attract the positively charged ions. The mechanism of the adsorption of copper and nickel ions on nanocellulose can be derived from this observation thanks to the information provided.

#### 3.1.4. BET Surface Area

**Figure 6:**
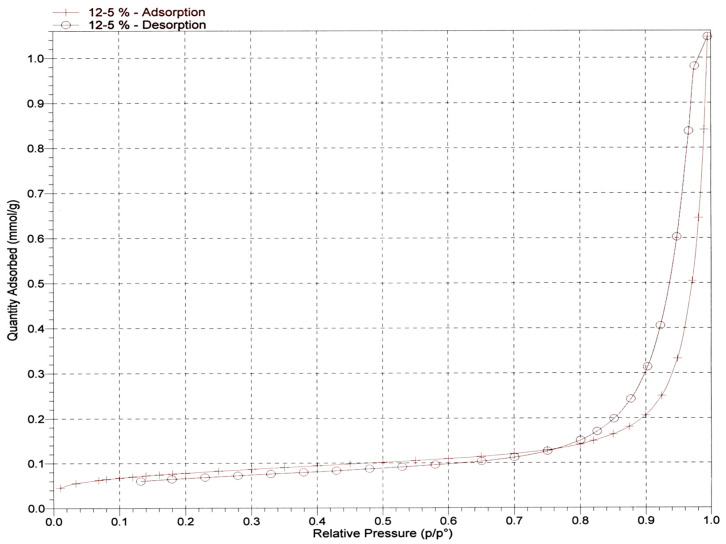
Nitrogen adsorption/desorption isotherms.

Scientists can determine the size of pores on test samples by employing a surface area analyzer, which measures the gas absorption capability of the samples. The effectiveness of a material in eliminating metal ions can be estimated by assessing the size of its pores during the adsorption procedure. This is because materials with larger pores tend to adsorb higher concentrations of metal ions present in the wastewater during the purification process. Adsorbing a gas onto the adsorbent that has a known molecular cross-sectional area, such as nitrogen dioxide (N_2_), allows for the measurement of the characteristics of the adsorbent, such as its pore size. Utilizing the Brunauer–Emmett–Teller (BET) and Barret, Joyner, and Halenda concepts in conjunction with gas adsorption-desorption techniques is an effective method for determining the porous material parameters of a material (BJH). In this step of the process, the pore size of the prepared nanocellulose is determined by using micrometric ASAP-2020 to estimate the distribution of pore sizes throughout the nanocellulose. In order to prepare the nanocellulose for testing, it was degassed in a vacuum at a temperature of 80 degrees for 18 h. The pore size of nanocellulose was measured using a technique called nitrogen adsorption/desorption, which was carried out at 77 kelvin (see Figure 6 for more details). For the nitrogen adsorption and desorption isotherms, the Brunauer, Emmett, and Teller (BET) method was utilized. This allowed for the collection of data that could be used to determine the pore size of the nanocellulose. Table 1 contains a listing of the results that were obtained from the isotherms.

### 3.2. The Effect of pH on Adsorption of Copper and Nickel Ions on Nanocellulose

**Figure 7:**
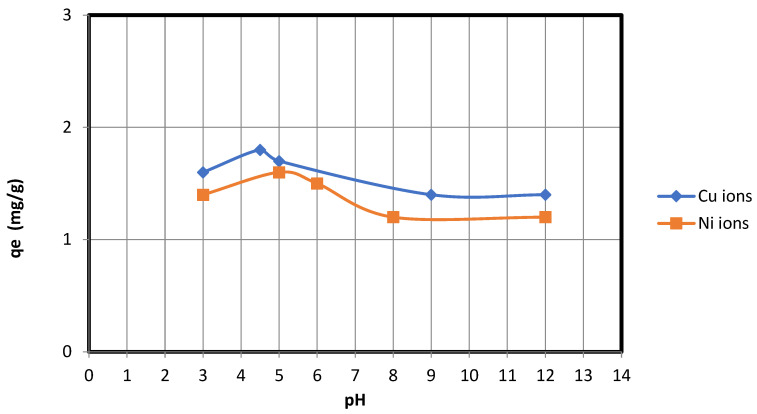
pH vs. qe for adsorption of Cu and Ni ions on nanocellulose.

pH is a crucial factor in adsorption processes. The pH also facilitates electrostatic interactions between sorbent materials and the ions to be adsorbed, making it a crucial control parameter for adsorption processes. In order to select the most appropriate conditions for future adsorption experiments, the effect of pH on the copper and nickel ions’ adsorption capacity and removal efficiency for nanocellulose was evaluated over the pH range of 2–12 (Figure 7). The effect of changing the pH from 2 to 12 is that NaOH (0.5 M) increased the pH value, whereas HCl (0.5 M) decreased the pH. As shown in Figure 7, the maximum adsorption of copper ions on nanocellulose occurs around pH = 4.5, while the maximum adsorption of nickel ions on nanocellulose occurs around pH = 5. This is due to the electrostatic attraction between the negative charges present in the nanocellulose and the positive charges found on the copper and nickel ions.

### 3.3. The Amount of the Adsorption of Copper and Nickel Ions on the Nanocellulose

**Figure 8:**
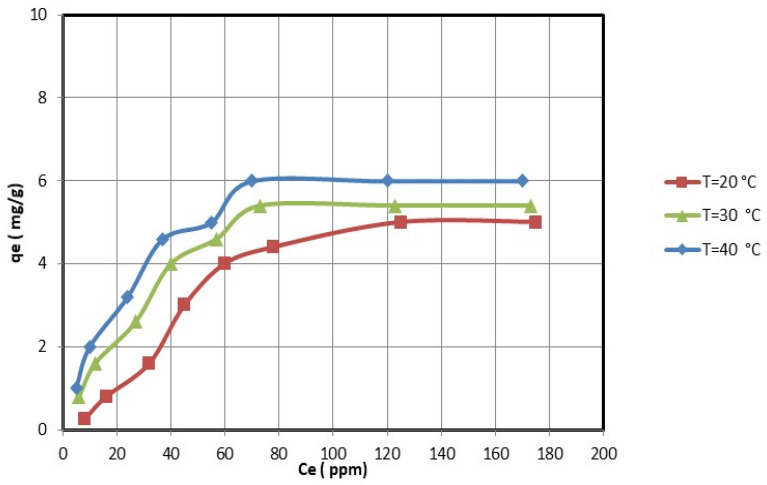
Adsorption of Ni ions on nanocellulose at different temperature levels.

**Figure 9:**
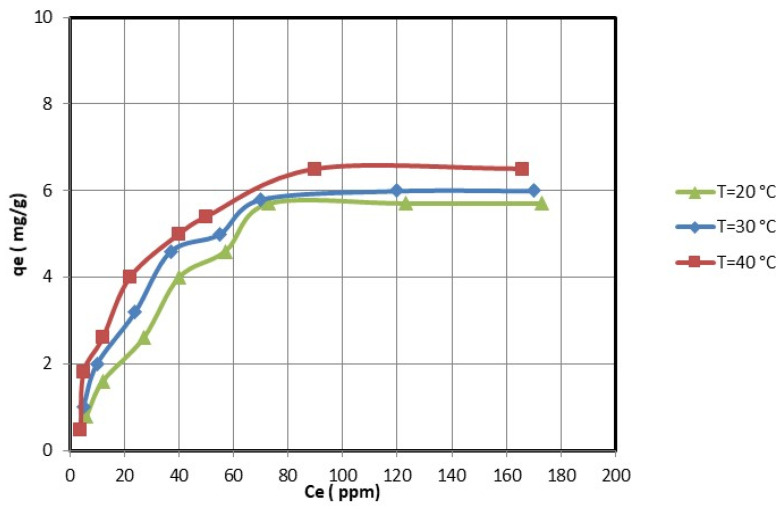
Adsorption of Cu ions on nanocellulose at different temperature levels.

For the optimal adsorption conditions (i.e., pH = 5 for nickel ions and pH = 4.5 for copper ions; 2 h; 0.25 g of nanocellulose per 50 mL of copper and nickel ions solution), the effect of the initial copper and nickel concentrations was studied. The equilibrium adsorption experiments were determined by varying the initial concentrations of copper and nickel ions. Figure 8 and Figure 9 show that the equilibrium adsorption isotherm curves for three different temperatures were obtained by plotting the amount of copper and nickel ions adsorbed on nanocellulose versus the equilibrium concentration of copper and nickel ions in the solution. The maximum adsorption capacity of copper ions on nanocellulose at room temperature (20 °C) was 5.7 mg/g. In comparison, the maximum adsorption capacity of nickel ions on nanocellulose at room temperature (20 °C) was 5 mg/g.

The electrostatic attraction between these negative sites and the copper and nickel ions causes the adsorption of these ions on the negative sites of the nanocellulose. In addition, the maximum adsorption amounts of copper and nickel ions on nanocellulose at room temperature (20 °C) were 5.7 mg/g and 5 mg/g, respectively. This is reasonable given that the atomic size of Cu is 128 pm compared to Ni’s 200 pm.

### 3.4. The Effect of Temperature on Adsorption of Copper and Nickel Ions on the Nanocellulose

The effect of temperature on the adsorption of copper and nickel ions by nanocellulose was studied at temperatures ranging from 20 °C to 40 °C. The adsorption capacities increase as the temperature rises, as shown in Figure 8 and Figure 9. Thus, the copper and nickel ion adsorption process of nanocellulose was endothermic. Therefore, increasing the adsorption temperature of copper and nickel ions on nanocellulose was favorable.

### 3.5. Fixed-Bed Adsorber Axial Pore Diffusion Model

To predict breakthrough curves when axial dispersion, external mass transfer, and pore diffusion control adsorption kinetics in a fixed bed, the dimensionless column mass balance equations and the equations describing the pore diffusion of the metal ions into the nanocellulose at each column location were solved numerically at the same time [22,23,24]:

(1) $$\frac{\partial C_{t}^{*}}{\partial t^{*}}=\gamma_{1} [\frac{1}{P_{e}}\left(\frac{\partial^{2}C_{{}_{t}}^{*}}{\partial z^{*2}}\right)-\frac{\partial C_{{}_{t}}^{*}}{\partial z^{*2}}-\beta_{1}\left(C_{{}_{t}}^{*}-C_{{}_{s}}^{*}\right)]$$

The initial and boundary conditions are:

(2) $$C_{t}^{*}=0\;\;\;\;\;\;\;\;\;\;\;\;\;\;\;\;\;\;\;\;\;\;\;\;\;\;\;\;\;\;\;\mathrm{for}\;t^{*}=0,\;z^{*}\geq 0$$

(3) $$\frac{\partial C_{t}^{*}}{\partial z^{*}}=-P_{e}\left(1-C_{t}^{*}\right)\;\;\;\;\;\;\;\;\;\;\;\;\;\mathrm{for}\;z^{*}=0,\;t^{*}\geq 0$$

(4) $$\frac{\partial C_{t}^{*}}{\partial z^{*}}=0\;\;\;\;\;\;\;\;\;\;\;\;\;\;\;\;\;\;\;\;\;\;\;\;\;\;\;\;\;\;\;\;\;\mathrm{for}\;z^{*}=1,\;t^{*}\geq 0$$

where

t = the actual time

$t^{*}=$ dimensionless time $=\frac{D_{p}\cdot t}{R_{p}^{2}}$

$\gamma_{1}=\frac{u_{s}\cdot R_{p}^{2}}{\varepsilon_{b}\cdot L\cdot D_{p}}$

$\beta_{1}=\frac{3\cdot k_{f}\cdot L\cdot \left(1-\varepsilon_{b}\right)}{u_{s}\cdot R_{p}}$

$P_{e}=\frac{L\cdot u_{s}}{D_{ax}}$

(5) $$\frac{\partial C_{t}^{*}}{\partial z^{*}}=0\;\;\;\;\;\;\;\;\;\;\;\mathrm{for}\;z^{*}=1,\;t^{*}\geq 0$$

(6) $$C^{*}=0\;\;\;\;\;\;\;\;\;\;\;\;\mathrm{for}\;t^{*}=0,\;0\leq r^{*}\leq 1$$

(7) $$\frac{\partial C^{*}}{\partial r^{*}}=0\;\;\;\;\;\;\;\;\;\;\;\;\mathrm{for}\;r^{*}=0,\;$$

(8) $$B_{i}\left(C_{t}^{*}-C^{*}\right)=\frac{\partial C^{*}}{\partial r^{*}}\;\;\;\mathrm{for}\;r^{*}=1$$

where $\beta \left(C^{*}\right)=\frac{1}{ [\varepsilon_{p}+\left(\frac{\rho_{p}\cdot q_{ref}}{C_{in}}\right)\left(\frac{1+b\cdot C_{in}}{{\left(1+b\cdot C_{in}\cdot C^{*}\right)}^{2}}\right)]}$, $C^{*}=\frac{C}{C_{in}}$, $C_{t}^{*}=\frac{C_{t}}{C_{in}}$ $q_{ref}=\frac{K\cdot C_{in}}{1+b\cdot C_{in}}$, $r^{*}=\frac{r}{R_{P}}$, $B_{i}=\frac{k_{f}\cdot R_{p}}{D_{p}}$ and $Z^{*}=\frac{Z}{L}$.

### 3.6. Numerical Solution of the Non-Linear Fixed-Bed Adsorption Systems

**Figure 10:**
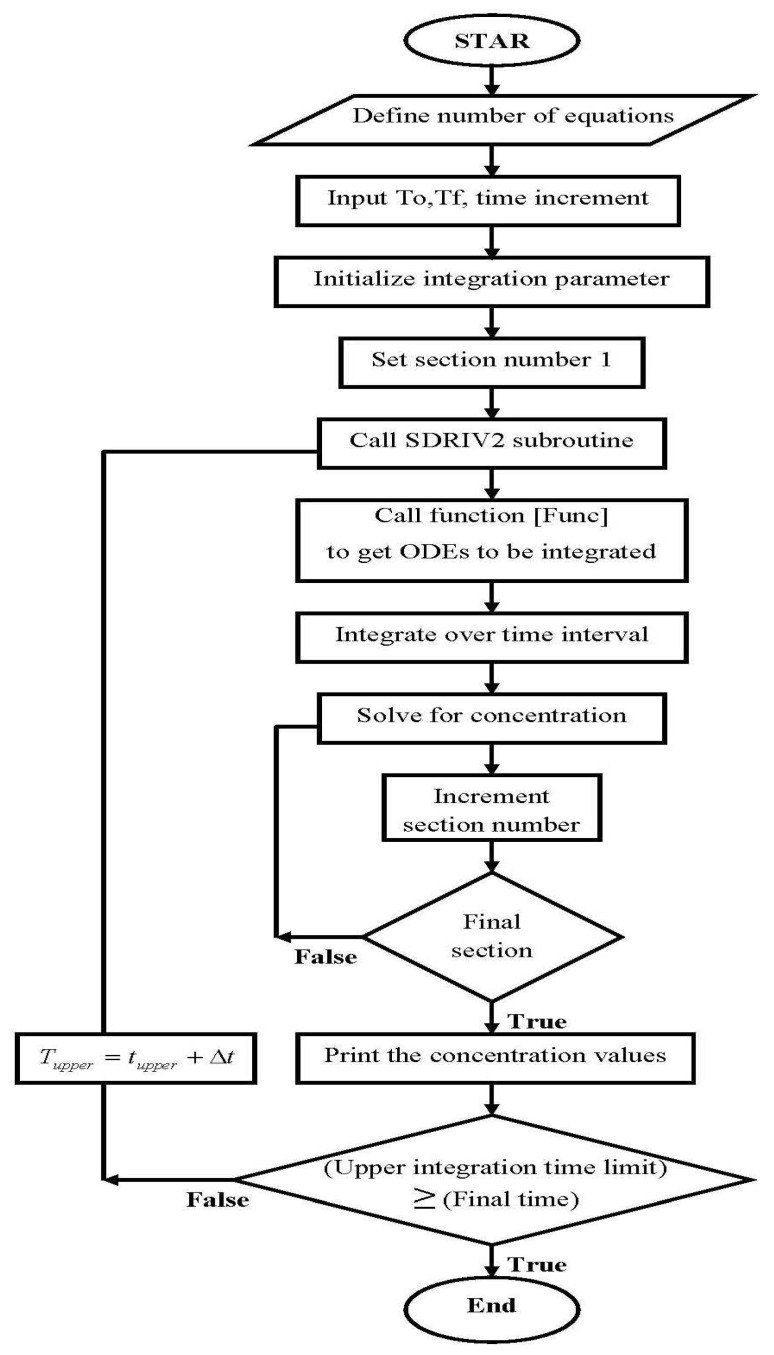
Flowchart for axial pore fixed-bed model.

The method of lines is a numerical method used to solve partial differential equations (PDEs). By using a finite-difference scheme to divide the spatial derivatives, the PDE is turned into a system of ordinary differential equations (ODEs). Specialized algorithms such as the Adam and Gear methods can then be used to solve the ODE. The column mass balance and pore diffusion equations for nanocellulose-using sorbents were solved using the line method. The subroutine SDRIV2 then solves these ODEs with Adam’s non-stiff ODE method and Gear’s stiff ODE method [25]. Figure 10 depicts the calculation procedure’s flowchart.

#### Estimation of the External Mass Transfer Coefficient and Axial Dispersion Coefficient for the Fixed Bed

The external mass transfer coefficient for the packed bed column, *k_f_* can be obtained using J-factor as follows [26]:

(9) kf=JD·usSc23

The axial dispersion coefficient, $D_{ax}$, is obtained by [15]:

(10) $$\frac{D_{ax}\cdot \rho_{f}}{\mu_{f}}=\frac{R_{e}}{0.2+0.011R_{e}^{0.48}}$$

For:

$$R_{e}=10^{-3}-10^{3}$$

### 3.7. Comparison of Prediction and Experimental Data of Axial Dispersion Pore Diffusion Model in Packed Bed

This study used a fixed-bed adsorber axial pore diffusion model to simulate and predict the breakthrough curves for the adsorption of copper and nickel ions on prepared nanocellulose biosorbents in a fixed-bed system. Axial pore-diffusion models are mathematical representations of the transport of a substance through the pores of a solid material. Typically, these models predict the breakthrough curves in fixed-bed adsorbers, representing the amount of a substance that has been adsorbed over time.

The model was developed based on a combination of partial differential equations for pore diffusion and copper and nickel mass balances in a fixed bed. To obtain the final results, it was necessary to determine several variables, including the axial dispersion and mass transfer coefficients.

In these models, the adsorption rate is assumed to be controlled by external mass transfer and pore-diffusion resistances. The line method can change the partial differential equations that describe the fixed-bed system into ordinary differential equations. The finite difference scheme can break up the spatial derivatives into smaller pieces. Then, these ODEs can be numerically solved to predict breakdown curves.

So, the axial dispersion coefficient was used to fit the experimental data to the axial pore diffusion model. This model considers how pore diffusion affects the adsorption process and how solutes move through the pores of the adsorbent. To obtain the breakthrough curve, the J-factor was used to figure out the mass transfer coefficient, which describes the mass transfer rate between the solid and liquid phases. The initial slope of the breakthrough curve, which represents the initial rate of adsorption, is a good indicator of the rate constant.

**Figure 11:**
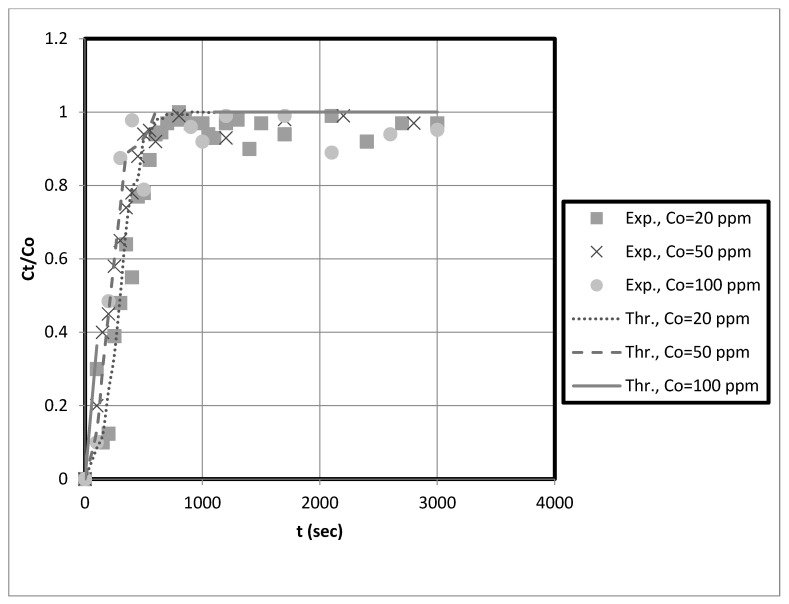
Axial pore diffusion model fit of the fixed-bed breakthrough curve for copper at different initial solution concentrations. Conditions: h = 0.05 m, us = 0.00473 m/s, kf = 0.000173 m/s, Dp = 2 × 10^−9^, Dax = 3.53 × 10^−6^ m^2^/s, eb = 0.42, r = 0.000175.

**Figure 12:**
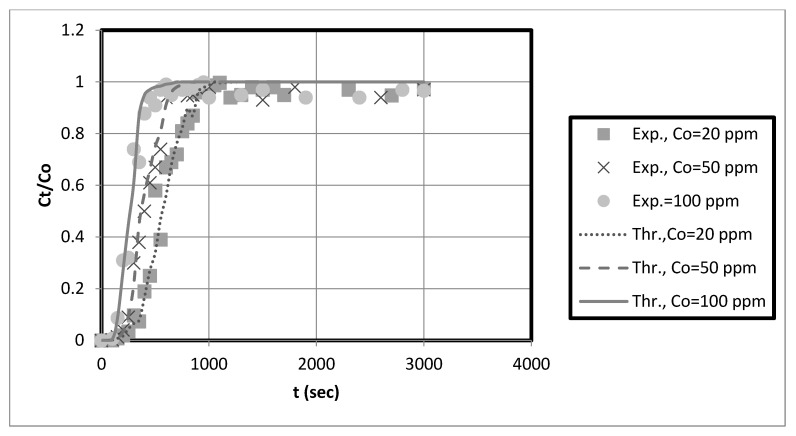
Axial pore diffusion model fit of the fixed-bed breakthrough curve for copper at different initial solution concentrations. Conditions: h = 0.1 m, us = 0.00473 m/s, kf = 0.000173 m/s, Dp = 2 × 10^−9^, Dax = 3.53 × 10^−6^ m^2^/s, eb = 0.42, r = 0.000175.

**Figure 13:**
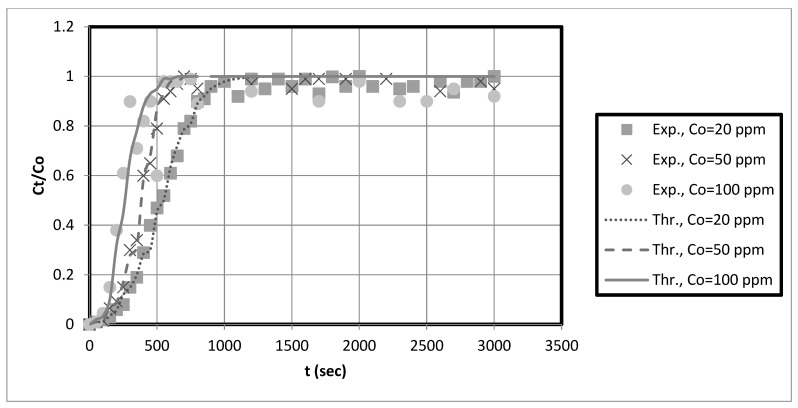
Axial pore diffusion model fit of the fixed-bed breakthrough curve for a nickel at different initial solution concentrations. Conditions: h = 0.05 m, us = 0.00473 m/s, kf = 0.000252 m/s, Dp = 2 × 10^−9^, Dax = 3.53 × 10^−6^ m^2^/s, eb = 0.42, r = 0.000175.

**Figure 14:**
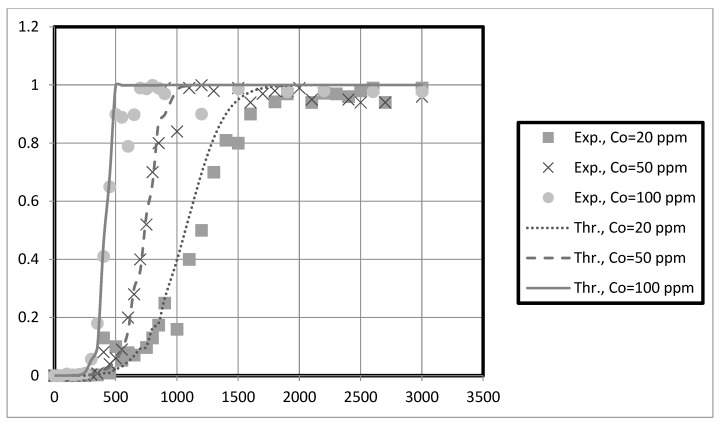
Axial pore diffusion model fit of the fixed-bed breakthrough curve for a nickel at different initial solution concentrations. Conditions: h = 0.1 m, us = 0.00473 m/s, kf = 0.000252 m/s, Dp = 2 × 10^−9^, Dax = 3.53 × 10^−6^ m^2^/s, eb = 0.42, r = 0.000175.

The experimental breakthrough data are compared to the external mass transfer pore diffusion model predictions, as shown in Figure 11, Figure 12, Figure 13 and Figure 14. The model is employed to forecast breakthrough curves in fixed-bed adsorbers. As demonstrated by the breakthrough curve, the model is applicable to accurately describing the adsorption process in the fixed-bed system. Typically, this is determined by comparing predicted breakthrough curves to experimental data to determine how well model predictions match observed behavior. A model is suitable for a given system if it can accurately predict the breakthrough curve. The data also revealed that the earlier the breakthrough occurred, the lower the bed height.

Furthermore, it was observed from these results that the earlier the breakthrough occurred, the higher the initial solution concentration. Futalan and Wan [27] observed the same behavior, noting that increased bed height resulted in more time for breaking through and exhaustion. Nevertheless, the fixed-bed system achieved a breakthrough and was depleted more rapidly as the initial concentration increased. Other researchers utilizing fixed-bed systems have discovered that at high initial concentrations, their systems reach breakthrough and depletion rapidly [14,27]. Due to the high initial concentration, the bed will become saturated more quickly.

The predicted breakpoints are compared to the experimental breakpoints in Table 2. The model was found to predict breakout points accurately. Consequently, the breakthrough point is a crucial indicator of the column’s performance in industrial applications. Table 2 reveals that the breakthrough point (3.33 at 10 cm bed depth) is greater than the breakthrough point (1.58 at 5 cm bed depth) when the initial concentration of the test solution is 20 mg/L, indicating that nanocellulose has a greater capacity to remove copper from the system. Additionally, for Ni, the breakthrough point (9.33) at 10 cm bed depth is more significant than that at 5 cm bed depth for an initial test solution concentration of 20 mg/L. Other scholars’ research that is similar to ours supports our findings [28]. They discovered that with increasing bed height, breakthrough and exhaustion times increased. In addition, the breakthrough point falls at bed height (e.g., 10 cm) as solution concentration rises. This reduces the time required to eliminate contaminants. Other researchers utilizing fixed-bed systems have discovered that at high initial concentrations, their systems rapidly reach breakthrough and exhaustion [14,27].

Funtan [27] conducted a similar study and found that raising the height of the bed increased both breakthrough and exhaustion. As the layer height increases, the breakthrough curve becomes less steep. Furthermore, we observed that the breakthrough curve shifted from left to right with increasing bed height, resulting in longer breakthrough and exhaustion times. Compared to the curve for high bed heights, the curve for low bed heights is steeper. The higher the bed height and the more adsorbent in the fixed bed, the more binding sites are available for adsorption during metal ion removal. In addition, the residence time of the feed water in the adsorbent bed is increased. The higher the bed height, the longer the contact time between the metal ions and the sorbent particles. Therefore, metal ions have sufficient time to transfer inside the adsorption bed for adsorption [27]. The breakthrough curve becomes steeper as the initial concentration increases, indicating rapid saturation of the fixed bed. The results in Table 2 suggest that the packed bed saturates faster, resulting in faster breakthrough and depletion times at high metal concentrations. Saturation and breakthrough times are faster as the initial metal concentration increases.

Overall, the fixed-bed adsorber axial pore diffusion model provided an excellent fit to the experimental data. In addition, the results showed that the model could accurately simulate the actual case for heavy metal removal using nanocellulose as an adsorbent.

The significance of this study lies in its ability to develop environmentally friendly and efficient techniques for treating industrial wastewater containing varying quantities of heavy metals. The study results demonstrate that prepared nanocellulose exhibits excellent adsorption capacity for removing copper and nickel ions from wastewater. The study has significant practical implications for developing sustainable solutions for wastewater treatment, especially in industries such as mining, metallurgy, and electroplating. Furthermore, this study’s findings can help reduce the environmental impact of these industries and improve the overall quality of water resources.

Nanocellulose is an exciting material with much potential for use in health and environmental fields, especially in cleaning toxic metals out of water. Additionally, its unique physical properties, such as high biodegradability, biocompatibility, and low cytotoxicity, have attracted much attention in many fields. Unlike inorganic nano-adsorbents, cellulose nanofibrils possess low genotoxicity and high biodegradability [29]. As a result, nanocellulose is widely considered a safe and biocompatible material that poses minimal risk to human health and the environment.

Nanocellulose composites can remove copper and nickel ions from wastewater and are considered safe for human consumption. This is because the material is biocompatible, biodegradable, and non-toxic. However, ensuring proper synthesis, processing, and purification of the composite is essential to prevent water contamination. If contamination is found, the particles can be removed from the water with physical methods such as filtration, centrifugation, or sedimentation.

Moreover, chemical treatments, such as coagulation and flocculation, can enhance the removal efficiency of the composite further. Therefore, it is crucial to employ appropriate purification methods to guarantee the safety of the water for human consumption. With these precautions in place, using nanocellulose composites to remove toxic metals from water bodies holds great promise for ensuring a healthier and safer environment.

The performance of nanocellulose prepared from waste palm leaves was comparable to that of the adsorbents reported in the literature [14,15,16,22,28,30], as shown in Table 3. The production of nanocellulose is very promising, the cost of waste palm leaves is low, and the material is obtained locally, which makes it a very cheap material. Other sorbents in Table 3 were also made from natural materials. Based on this result, the nanocellulose produced in this work showed competitive performance compared to the sorbents in Table 3. In general, nanocellulose offers several benefits as an adsorbent, such as being sustainable, cost-effective, renewable, and efficient in removing heavy metal contaminants. However, certain drawbacks must be taken into account. For instance, nanocellulose may lack selectivity for specific heavy metal ions and may remove other ions present in wastewater, which could reduce the overall effectiveness of the treatment process.

## 4. Conclusions

This paper presents a mathematical approach to simulate and predict breakthrough curves for the adsorption of copper and nickel ions from wastewater onto prepared nanocellulose biosorbents using a fixed-bed system. The study aimed to address potential sources of environmental hazards, such as heavy metals in wastewater and their disposal on land.

The mathematical model is based on a combination of partial differential equations for pore diffusion and copper and nickel mass balances in a fixed bed. Different experimental parameters, including bed height (5 cm, 10 cm) and initial concentration (20–100 mg/L), were tested with the model. It was found that these parameters influenced the shape of the bedtime and breakthrough curves.

The breakthrough point increased with bed height at an initial concentration of 20 mg/L, allowing more time to remove pollutants. The maximum adsorption amounts of copper and nickel ions on nanocellulose at room temperature (20 °C) were 5.7 mg/g and 5 mg/g, respectively. At bed heights (e.g., 10 cm), the breakthrough point decreased with increasing solution concentration, reducing the time required to remove contaminants.

The difference in error between the experimental and model data provided a clue that the model developed in this study can accurately simulate the actual case for heavy metal removal. Overall, the new model can closely predict the actual situation for the adsorption of Cu and Ni from wastewater using nanocellulose as a readily available adsorbent under varying experimental conditions. Furthermore, the breakthrough curves calculated from the fixed-bed axial pore diffusion model correlated well with the experimental data. Although the paper did not explicitly focus on membranes, the research highlights the potential of nanocellulose as a valuable material for membrane technology.

## Figures and Tables

**Table 1:** The average pore size.

Element	Nanocellulose
Average pore width (4V/A by BET), nm	11.13

**Table 2:** Experimental results.

Metal Type	Initial Concentration, Co, ppm	Bed Height, z, cm	The Breakthrough Point, tb (min)	The Breakthrough Point, tb (min)	Error (%)
			Experiment	Predicted by the Model	
Copper	20	5	1.580	1.667	5.22
		10	3.330	3.830	13.05
	50	5	0.916	0.816	12.25
		10	1.830	1.950	6.15
	100	5	0.730	0.670	8.96
		10	1.800	1.660	8.43
Nickel	20	5	5.167	5.000	3.34
		10	9.330	10.000	6.70
	50	5	3.280	3.770	12.90
		10	7.000	8.330	15.97
	100	5	2.300	2.700	14.81
		10	4.500	4.86	7.40

**Table 3:** A comparison of prepared nanocellulose with the other reported literature results.

Ref.	Adsorbent	Solution	T (°C)	Removal	Capacity
	Type			Percent%	mg/g
[14]	Fe_2_O_3_ carbon foams	Copper ions	25		12
		Nickle ions	25		9.5
[15]	natural zeolites	Copper ions	25	14.55	
[16]	Chitosan magnetic beads	Nickle ions	25	79.5	
[22]	bacterial biomass	Nickle ions	25		5.7
[22]	activated carbons from organic material	Nickle ions	25		5.8
[28]		Copper ions	25		
	Palm kernel shells	Nickle ions	25	97 55	1.581 0.130
[30]	African palm charcoal	Copper ions Nickle ions	25 25	92 90.4	
This work	Nanocellulose	Copper ions Nickle ions	25 25	60 53	5.7 5

## Data Availability

Not applicable.

## Abbreviations

b	Langmuir isotherm parameter, (liter/mg).
C	metal concentration inside the pores of the adsorbent particle, (ppm).
$C_{e}$	Equilibrium metal solution concentration, (ppm).
$C_{0}$	initial metal solution concentration, (ppm).
$C_{s}$	metal solution concentration on the outer adsorbent surface, (ppm).
$C_{t}$	metal solution concentration at any time t, (ppm).
$D_{ax}$	axial dispersion coefficient (cm^2^/s).
$d_{p^{}}$	average particle size, (micrometer).
$D_{P}^{}$	effective pore diffusion coefficient, (cm^2^/s).
$D_{c}^{}$	internal diameter of the bed column, (cm)
$J_{D}$	the dimensionless mass transfer factor.
K	Langmuir isotherm parameter, (liter/g).
$k_{f}$	external mass transfer coefficient, (cm/s).
m	mass of adsorbent.(g).
$m_{s}$	mass of adsorbent per metal solution volume, g/cm^3^.
q	adsorbed metal concentration as a function of position in adsorbent particle, (mg/g).
$q_{avg.}$	average adsorbed metal concentration in the adsorbent particle, (mg/g).
$q_{e}$	equilibrium metal adsorbed concentration, (mg/g).
r	radius along the adsorbent particle, 0 ≤ r ≤ Rp, (cm).
$R_{p}^{}$	radius of the adsorbent particle, (cm).
$R_{e}^{}$	Reynolds number, dimensionless.
$S_{c}^{}$	Schmidt number, dimensionless.
t	time (s)
$u_{s}$	superficial velocity (cm/s).
V	metal solution volume, (liter).
z	length along the bed, 0 ≤ z ≤ L, (cm).
Greek letters	
$\varepsilon_{{}_{b}}$	bed porosity.
$\varepsilon_{{}_{p}}$	Particle porosity.
$\mu_{f}$	solution viscosity (g/cm·s).
$\rho_{{}_{p}}$	particle density, (g/cm^3^).
$\rho_{{}_{s}}$	solid density, (g/cm^3^).

## References

[1] Hegazi H. (2013). Removal of heavy metals from wastewater using agricultural and industrial wastes as adsorbents. HBRC J..

[2] Esmaeilia H., Tamjidib S., Abed M. (2020). Removal of Cu(II), Co(II), and Pb(II) from synthetic and real wastewater using calcified Solamen Vaillanti snail shell. Desalination Water Treat..

[3] Khamseh A., Amini Y., Shademan M., Ghazanfari V. (2023). Intensification of thorium biosorption onto protonated orange peel using the response surface methodology. Chem. Prod. Process Model..

[4] Onundi Y., Mamun A.A., Ahmed M. (2010). Adsorption of copper, nickel and lead ions from synthetic semiconductor industrial wastewater by palm shell activated carbon. Int. J. Environ. Sci. Technol..

[5] Chang-GuLeea C., SoonjaeLeeb S., Parka J., Park C., Lee Kim S., An B., Yun S., Lee S., Choia J. (2017). Removal of copper, nickel and chromium mixtures from metal plating wastewater by adsorption with modified carbon foam. Chemosphere.

[6] Abdulrazak S., Hussaini K., Sani H. (2017). Evaluation of removal efficiency of heavy metals by low-cost activated carbon prepared from African palm fruit. Appl. Water Sci..

[7] Tahya C., Cornelia M., Siregar T., Taipabu M. (2022). Adsorption of Copper Ion from Acidic Wastewater by Local Natural Zeolite. Walisongo J. Chem..

[8] Khamseh A., Ghorbanian S. (2018). Experimental and modeling investigation of thorium biosorption by orange peel in a continuous fixed-bed column. J. Radioanal. Nucl. Chem..

[9] Hajeeth T., Gomathi T., Sudha N. Adsorption of Copper (Ii) and Nickel (Ii) Ions from Metal Solution using Graft Copolymer of Cellulose Extracted from The Sisal Fiber with Acrylonitrile Monomer. Proceedings of the International Conference on Advanced Nanomaterials & Emerging Engineering Technologies (ICANMEET-20/3).

[10] Lombardo S., Thielemans W. (2019). Thermodynamics of adsorption on nanocellulose surfaces. Cellulose.

[11] Bertsch P., Fischer P. (2020). Adsorption and interfacial structure of nanocelluloses at fluid interfaces. Adv. Colloid Interface Sci..

[12] Madivoli E., Kareru P., Gachanja A., Mugo S., Murigi M., Kairigo P., Kipyegon C., Mutembei J., Njonge F. (2016). Adsorption of Selected Heavy Metals on Modified Nano Cellulose. Int. Res. J. Pure Appl. Chem..

[13] Noman E., Al-Gheethi A., Maya R., Mohamed S., Al-Sahari M., Hossain M., Vo D., Naushad M. (2022). Sustainable approaches for nickel removal from wastewater using bacterial biomass and nanocomposite adsorbents: A review. Chemosphere.

[14] Himanshu P. (2020). Batch and continuous fixed bed adsorption of heavy metals removal using activated charcoal from neem (*Azadirachta indica*) leaf powder. Sci. Rep..

[15] Mckay G., Bino M. (1990). Simplified Optimization Procedure for Fixed Bed Adsorption System. Water Air Soil Pollut..

[16] Sekaran G., Shanmugasundaram K.A., Mariappan M., Raghavan K.V. (1995). Utilization of Solid Waste Generated in Leather Industry for Removal of Dye in Aqueous Solution. Indian J. Chem. Technol..

[17] Saud A., Saleem H., Zaidi J. (2022). Progress and Prospects of Nanocellulose-Based Membranes for Desalination and Water Treatment. Membranes.

[18] Tan H., Ooi B., Leo C. (2020). Future Perspectives of Nanocellulosebased Membrane for Water Treatment. J. Water Process Eng..

[19] Liu p., Zhu C., Mathew A. (2019). Mechanically Robust High Flux Graphene Oxide-nanocellulose Membranes for Dye Removal from Water. J. Hazard. Mater..

[20] Jaffar S., Saallah S., Misson M., Siddiquee S., Roslan J., Saalah S., Lenggoro W. (2022). Recent Development and Environmental Applications of Nanocellulose-Based Membranes. Membranes.

[21] Rana K., Gupta K., Saini K., Voicu I., Abdellattifaand H., Thakur K. (2021). Water desalination using nanocelluloses/cellulose derivatives based membranes for sustainable future. Desalination.

[22] Juang R.S., Tseng R.L., Wu F.C., Lee S.H. (1997). Adsorption Behavior of Reactive Dyes from Aqueous Solutions on Chitosan. J. Chem. Technol. Biotechnol..

[23] Stuar F.X., Camp D.T. (1973). Solution of The Fixed Bed Physical Adsorption Problem With Two Significant Rate Controlling Steps. AIChE Symp. Ser..

[24] Brauch V., Schlunder E.U. (1975). The Scale- Up of Activated Carbon Columns for Water Purification, Based on Results from Batch Tests-II. Chem. Eng. Sci..

[25] Moon H., Lee W.K. (1983). Intraparticle Diffusion in Liquid—Phase Adsorption of Phenols With Activated Carbon in Finite Batch Adsorber. J. Coll. Interface Sci..

[26] Schiesser W.E. (1991). The Numerical Method of Lines: Integration of Partial Differential Equations.

[27] Futalan C., Wan M. (2022). Fixed-Bed Adsorption of Lead from Aqueous Solution Using Chitosan-Coated Bentonite. Int. J. Environ. Res. Public Health.

[28] Dinesha B.L., Hiregoudar S., Nidoni U., Ramappa K.T., Dandekar A.T., Ganachari S.V. (2022). Adsorption modelling and fixed-bed column study on milk processing industry wastewater treatment using chitosan zinc-oxide nano-adsorbent-coated sand filter bed. Environ. Sci. Pollut. Res. Int..

[29] Ramos-Vargas S., Huirache-Acuña R., Rutiaga-Quiñones J., Cortés-Martínez R. (2020). Effective lead removal from aqueous solutions using cellulose nanofibers obtained from water hyacinth. Water Supply.

[30] Futalan C.M., Kan C., Lourdes M., Dalida P. (2011). Nickel Removal from Aqueous Solution in Fixed Bed Using Chitosan-Coated Bentonite. Sustain. Environ. Res..

